# Temporal Dynamics of Visual Attention Allocation

**DOI:** 10.1038/s41598-019-40281-7

**Published:** 2019-03-06

**Authors:** Jongmin Moon, Seonggyu Choe, Seul Lee, Oh-Sang Kwon

**Affiliations:** Department of Human Factors Engineering, Ulsan National Institute of Science and Technology, Ulsan, 44919 Republic of Korea

## Abstract

We often temporally prepare our attention for an upcoming event such as a starter pistol. In such cases, our attention should be properly allocated around the expected moment of the event to process relevant sensory input efficiently. In this study, we examined the dynamic changes of attention levels near the expected moment by measuring contrast sensitivity to a target that was temporally cued by a five-second countdown. We found that the overall attention level decreased rapidly after the expected moment, while it stayed relatively constant before it. Results were not consistent with the predictions of existing explanations of temporal attention such as the hazard rate or the stimulus-driven oscillations. A control experiment ruled out the possibility that the observed pattern was due to biased time perception. In a further experiment with a wider range of cue-stimulus-intervals, we observed that attention level increased until the last 500 ms of the interval range, and thereafter, started to decrease. Based on the performances of a generative computational model, we suggest that our results reflect the nature of temporal attention that takes into account the subjectively estimated hazard rate and the probability of relevant events occurring in the near future.

## Introduction

When we see the surrounding environment, our eyes receive a huge amount of sensory input. Rather than processing all available sensory input with an equal level of depth, our visual system selects a specific subset that is supposedly more relevant to the tasks at hand, and processes that input in more detail than the rest^1,2^. This selective visual attention makes it possible for the brain to effectively respond to relevant visual signals while avoiding excessive neural activity to process all sensory input^3,4^.

**Figure 1:**
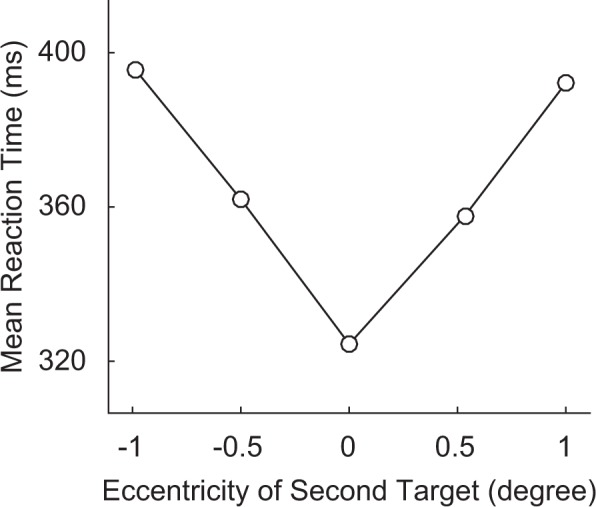
Mean reaction times as a function of eccentricities of a second target from a study by LaBerge and Brown^9^. A symmetric pattern of attentional changes across the spatial domain can be observed.

Selective visual attention is often allocated to a specific spatial position or objects^5,6^ at a specific temporal moment^7^. One of the natural questions regarding spatial and temporal properties of visual attention is how attention spreads out from a specific position and moment on which attention is focused. The characteristics of the spatial extension of selective attention are well documented. According to the gradient model, attentional resources are allocated in a gradient pattern, which means attention level is highest at the attended location, and symmetrically and gradually decreases away from the location^8,9^. For example, LaBerge and Brown^9^ presented two sets of horizontally arranged eleven characters in succession. Subjects were to determine if the target letter was included in both the first and second stimuli among flanker characters. The target letter in the first stimulus was located at the center of the horizontal array, which encouraged subjects to pay attention at the center. The key manipulation was the location of the target letter in the second stimulus, which was presented at random places. The reaction time to determine whether the targets appeared in both stimuli was fastest when the target of the second stimulus was presented at the center, and gradually increased as the distance of the second target from the center increased (Fig. 1). Results clearly showed that spatial attention gradually and symmetrically decreased from the focused point.

While the spatial extension of visual attention is relatively well understood, it is not clear how visual attention extends around the focal moment in time. It has been proposed that two distinct mechanisms are responsible for the temporal changes of attention. One is the exogenous attention driven by regular temporal rhythms, and the other is the endogenous attention modulated by conditional probability.

The temporal entrainment is a representative phenomenon that shows properties of the exogenous temporal attention^10^. In an experiment of temporal entrainment in visual tasks, subjects were asked to detect a visual target embedded in a stream of repeating visual stimuli with interstimulus intervals (ISI) less than 100 ms. Subjects detected a visual target better when the target is presented in sync with the repeatedly presented entraining stimuli, rather than when the target is presented out of sync. Results demonstrated that the temporal attention automatically induced by entraining stimuli modulates the sensitivity to a visual target^10–15^.

The endogenous temporal attention is modulated by the conditional probability of a target appearance at a given moment, which were commonly manipulated by the hazard rate or contingency between the symbolic cue and cue-target interval^16–24^. In studies testing the effects of the hazard rate on temporal attention, subjects were asked to respond as quickly as possible to a target^16–21^ or to identify a target accurately^23,24^ while the ISI between a cue and the target is randomly selected within a range of ISIs. It was found that both the reaction time^16–21^ and the perceptual processing speed decreased^23,24^ as the conditional probability of the target appearance at the moment increased. For example, subjects’ performances were better at longer ISIs when ISIs were distributed uniformly, because the hazard rate increased as ISI increased in such a condition. Moreover, it was reported that subjects’ performances tested in conditions with diverse distributions of ISIs reflect the shapes of the corresponding hazard rate^17–19,23^.

In the current study, we had subjects orient their attention to a certain point in time, and estimated the allocated attention at and around that focal point in time. Numerous studies have empirically shown that attention improves perceptual and motoric performance^5,25,26^. Attention improves contrast sensitivity^26–28^ and decreases reaction time to spatially or temporally cued stimulus^5,7,29^, which were accompanied by increased neural activity in the corresponding brain areas^7,29–32^. Although the dynamics of temporal attention can be estimated by either perceptual or motoric tasks, in case of a motoric task, it is difficult to disentangle the effects of visual attention and motor preparation on the resulting reaction times^32,33^. Therefore, we opted to use a perceptual task to estimate the temporal dynamics of visual attention. We used a five-second countdown to attract subjects’ attention on the temporal focal moment and presented a target stimulus at a random time around the moment. We measured contrast sensitivity to the target to estimate the amount of attention allocated.

If the exogenous attention mechanism dominantly determines the attention allocation, the contrast sensitivity would be highest at the focal moment, and gradually decreases as the interval between the focal moment and the target increases. This is because of the temporal oscillation of the stimulus-driven attention that peaks at the focal moment. Results will be similar to the V-pattern of attentional changes around an attended location that are observed in the spatial domain. On the other hand, if the endogenous attention dominantly determines the attention allocation, the contrast sensitivity would monotonically increase as the cue-target-interval increases. This is because the hazard rate monotonically increases when ISIs are distributed uniformly. Results showed that neither was the case. Attention allocation decreased faster after the attention-induced moment than before the attention-induced moment.

We ran two additional experiments to constrain possible explanations for the results. Experiment 2 ruled out the possibility that the observed pattern was due to biased time perception. Results of Experiment 3 showed that the pattern of temporal attention allocation was affected by the range of ISIs.

## Results

### Temporal dynamics of visual attention

**Figure 2:**
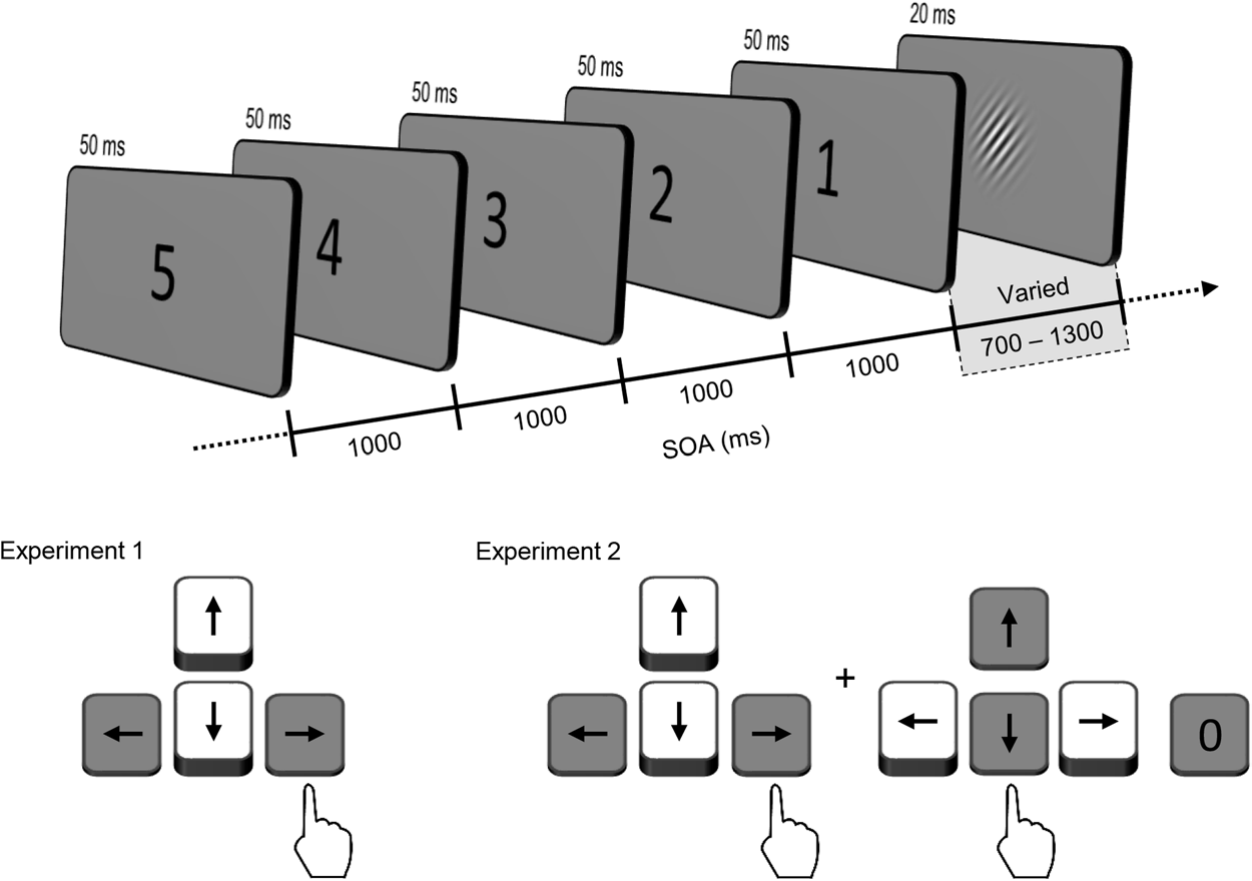
Schematic illustration of a sequence of events in a single trial in Experiment 1 and 2. The target stimulus, a tilted Gabor patch, was temporally cued by a five-second countdown. The interval duration between the final countdown and the target stimulus onset was randomly chosen from eight equally spaced points from 700 ms to 1300 ms. Subjects reported the orientation of the stimulus by pressing either a left arrow key (leftward tilt) or a right arrow key (rightward tilt). In Experiment 2, subjects performed a duration judgment task after reporting orientation. They indicated whether they perceived the interval between the final countdown and target onset as being shorter or longer than one second by pressing a down arrow key (shorter) or up arrow key (longer). Subjects pressed “0” key when they missed the target.

Subjects were presented with a Gabor stimulus that was temporally cued by a visual five-second countdown from number “5” to “1” (see Fig. 2). Each number was briefly presented for 50 ms in 1000 ms intervals. The interval duration between the final countdown (i.e. the number “1”) and the Gabor stimulus onset was randomly chosen from eight equally spaced points, from 700 ms to 1300 ms. Subjects were asked to report the orientation of the Gabor stimulus. We measured the contrast sensitivity to the target as a function of the interval duration.

**Figure 3:**
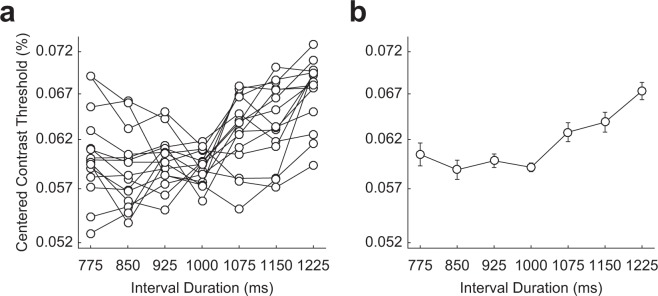
Centered contrast thresholds of (**a**) 15 subjects and (**b**) their means as a function of interval durations. Despite an equal probability of target appearance for every interval duration, contrast thresholds were significantly different depending on interval duration (*F*_6,84_ = 9.12, *p* < 0.001, ${\eta }_{{\rm{p}}}^{2}$ = 0.39). Contrast thresholds remained constant until one second had elapsed and increased afterward. The error bars represent standard errors of the means (*n* = 15).

Figure 3a shows centered contrast thresholds of the 15 subjects as a function of interval duration, and Fig. 3b shows averages of the centered contrast thresholds across subjects. We applied a moving average with a window size 3 to highlight the overall trends of temporal dynamics (see Methods for details). Even though the stimulus appeared with equal probability across interval durations, contrast thresholds systematically varied across interval durations. ANOVA results revealed a significant effect of interval duration on contrast threshold (*F*_6,84_ = 9.12, *p* < 0.001, ${\eta }_{{\rm{p}}}^{2}$ = 0.39). The systematic changes in contrast thresholds implied that subjects’ attention dynamically varied across interval durations.

If dynamic changes of temporal attention were mainly determined by the stimulus-driven oscillation^10–15^, the contrast threshold function (Fig. 3b) would form a V-shape, manifesting attentional allocation symmetrically, and gradually decreasing as it moves away from the focal point. If dynamic changes of temporal attention reflect the hazard rate, the contrast threshold would decrease as a function of interval durations, showing overall increase of temporal attention. However, the results were consistent with neither of the hypotheses. As shown in Fig. 3b, the threshold remained relatively constant until one second had elapsed and then increased afterward. Confirming this observation, pairwise comparison analysis between interval durations showed that the three contrast thresholds before 1000 ms were not significantly different from the threshold at 1000 ms (*p* > 0.34), whereas the three thresholds after 1000 ms were significantly higher than the threshold at 1000 ms (*p* < 0.01). Full pairwise comparison analysis results can be found in Table S1.

### Temporal attention is not determined by biased time perception

The results of Experiment 1 show that the dynamics of temporal attention do not manifest in symmetric patterns around the attention-induced point, one second. However, if subjects focused their attention at a moment earlier than the one second, and the attention decreased symmetrically around that moment, the observed pattern of attentional changes can emerge. It is possible that subjects perceived the duration of less than one second as one second, and, as a result, placed their focal attention at the time point perceived as one second. Experiment 2 was conducted to examine whether there was a perceptual bias in time estimation during the task. We asked subjects to judge whether the interval duration between the final countdown and target stimulus was shorter or longer than one second (Fig. 2). Two contrast levels were used (Experiment 2A and 2B) to examine the effect of stimulus contrast on time perception. The rest of the stimuli and procedure were identical to those of Experiment 1.

**Figure 4:**
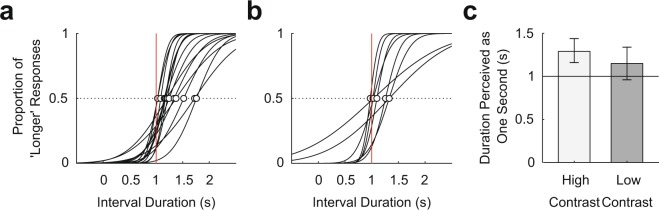
Results of Experiment 2. (**a**) Individual subjects’ psychometric function in Experiment 2A (low contrast; *n* = 15). The white circles represent the duration perceived as one second for each subject, and the vertical lines indicate an actual one second. (**b**) Individual subjects’ psychometric function in Experiment 2B (high contrast; *n* = 8). (**c**) The bars represent the population modes for the duration perceived as one second and the error bars represent 95% credible intervals. The horizontal line indicates an actual one second. The duration perceived as one second was significantly longer than an actual one second in Experiment 2A (mode = 1.29 s, 95% credible interval = [1.16, 1.44]). Similar trend was found in Experiment 2B (mode = 1.15 s, 95% credible interval = [0.96, 1.34]).

The results of Experiment 2A showed that the duration perceived as one second was significantly longer than a second (mode = 1.29 s, 95% credible interval = [1.16, 1.44]; Fig. 4a,c), implying that subjects felt that the time between the last countdown and the target stimulus went faster than reality. Consequently, subjects perceived the duration of one second to be shorter than a second, and durations longer than one second as one second. The durations perceived as one second in Experiment 2B were similar to those in Experiment 2A, although they were not significantly different from a second (mode = 1.15 s, 95% credible interval = [0.96, 1.34]; Fig. 4b,c).

The results of Experiment 2 showed that the duration perceived as one second was longer than an actual second. Thus, if subjects have paid attention to the moment of the perceived one second, the performance should have been better after the physical one second than before. The results of Experiment 1 showed opposite pattern. This result clearly rules out the possibility that asymmetry of attention allocation around the attention-induced moment (1 second) was caused by a biased time perception.

### Does time scale matter

The result of Experiment 1 largely deviates not only from the symmetric shape predicted by the stimulus-driven oscillation, but also from the prediction of the hazard rate that is often reported in studies using similar manipulation of interval durations^16–24^. We speculated that such inconsistency between the current results and the literature could be due to a relatively narrow range (600 ms) of interval durations used in Experiment 1 compared to existing studies (around 3 seconds). We tested whether a wider range of interval durations would yield a temporal pattern of contrast thresholds that differs from the results of Experiment 1. A new group of 15 subjects participated. Subjects were presented with a Gabor stimulus that was temporally cued by a visual countdown from two. The interval duration between the onset of the number “1” and the Gabor stimulus was randomly chosen from eleven equally spaced points ranging from 500 ms to 3000 ms.

**Figure 5:**
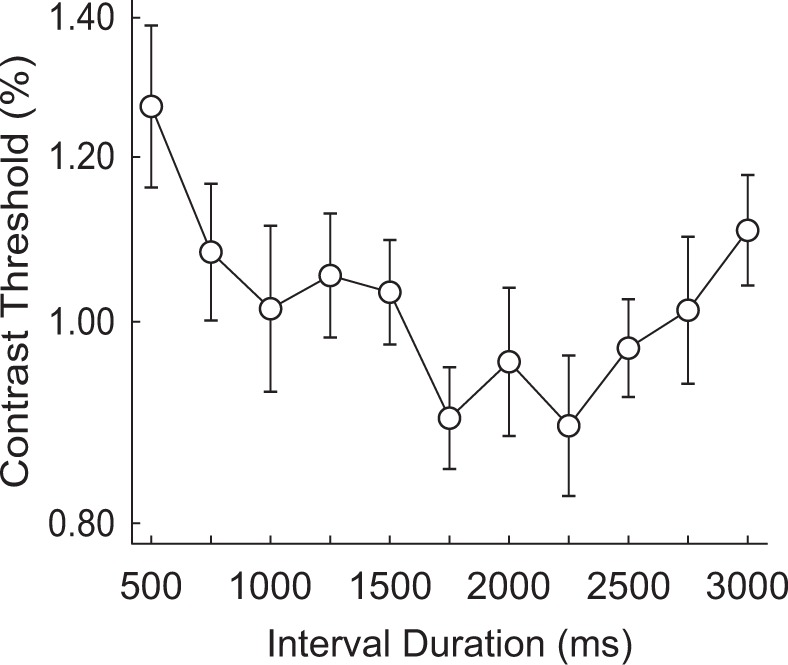
Results of Experiment 3. A significant effect of interval duration was again observed (*F*_10,140_ = 3.68, *p* < 0.001, ${\eta }_{{\rm{p}}}^{2}$ = 0.21), even with a wider range of interval durations than Experiment 1. The pattern was qualitatively different from that of Experiment 1, as contrast thresholds generally decrease as a function of interval durations following the hazard rate but increase near the end of the range. The error bars represent *SD* of the bootstrapped distribution.

Overall, contrast thresholds (Fig. 5) tend to decrease for most of the interval durations (≤2250 ms) and start to increase near the end of the interval range (>2250 ms). Results of ANOVA showed a significant main effect of interval durations (*F*_10,140_ = 3.68, *p* < 0.001, ${\eta }_{{\rm{p}}}^{2}$ = 0.21). Pairwise comparison between interval durations further revealed that subjects’ performances at 2250 ms was significantly better than their performances at not only the early parts (500 ms, 750 ms, 1250 ms, and 1500 ms) of the interval range (*p* < 0.03), but also the later part (3000 ms) of the interval range (*p* = 0.004). Full pairwise comparison analysis results can be seen in Table S2.

Results suggest that the time scale does matter. The contrast thresholds generally decreased across duration intervals, and an increase of the contrast thresholds was found only at the later part of the interval range. The overall decrease of contrast thresholds (i.e. increasing attention level) is qualitatively consistent with the prediction of hazard rate. However, the increase of thresholds at the later part remains to be explained.

### A generative model with a predictive attention

Then, what caused the observed results? A characteristic of temporal attention is that it is for future, and not for past events, and it is difficult to predict the exact moment of a future event. Therefore, visual temporal attention is better to be prepared before the event than after, in order to process the upcoming stimulus effectively. We developed a computational model to quantitatively evaluate the idea that attention levels at a given moment depend on the probability that a target will appear at that moment or in the near future.

When the interval duration between a cue and target varies randomly, the probability of a target appearing at time *t*, given that the target has not appeared until that moment, can be described by a hazard function^16–24^. We modeled the discrete uniform distributions of interval durations used in the experiments as a continuous uniform distribution demarcated by the minimum and maximum values of intervals used in the experiments. For a proper modeling of the current data, we considered an additional term reflecting that subjects may not notice the appearance of a target in about half the trials, as the contrast of the target stimulus in the current study was at around the threshold level. This term has not been considered in earlier studies, presumably because the stimuli used in those studies were too salient to miss. Taking into account the probability of missing the target, the probability of the target’s appearance at a given moment (i.e. hazard rate) can be formulated as

1 $$h(t)=\frac{f(t)}{1-kF(t)}$$

where *h*(*t*) is the probability of the target appearing at time *t*, *f*(*t*) is the probability density function of interval durations, $F(t)$ is the cumulative distribution function, ${\int }_{0}^{t}f(s)ds$, and *k* is given by $k=1-P(ND|A)$. Here, $P(ND|A)$ is the probability that the target is not detected after it appeared, which was set to 0.5 (i.e. threshold level).

The measurement noise^34^, which is unavoidable in human time perception, was modeled based on the assumption that the size of noise grows linearly with time^19,35,36^. Given a measurement *m*, the likelihood of *t* is denoted by $p(m|t)$. We set $p(m|t)$ as a Gaussian distribution with mean *t* and standard deviation *wt*, in which the coefficient of variation, *w*, is a free parameter characterizing the Weber fraction for time estimation.

2 $$p(m|t)=\frac{1}{\sqrt{2\pi {(wt)}^{2}}}{e}^{\frac{-{(m-t)}^{2}}{2{(wt)}^{2}}}$$

Note $p(m|t)$ given a fixed measurement *m* is not symmetric because of the Weber fraction. The hazard rate given a noise contaminated measurement *m* incorporating the likelihood of elapsed time, $p(m|t)$, and the hazard rates, $h(t)$, can be written as:

3 $$\hat{h}(m)={\int }^{}h(t)p(t|m,ND)dt=\frac{{\int }^{}f(t)p(m|t)dt}{{\int }^{}(1-kF(t))p(m|t)dt}$$

where $\hat{h}(m)$ denotes a subjective hazard function (see Supplementary Information for details). Since we were unable to track subjects’ measurement *m* on each trial, we integrated $\hat{h}(m)$ over *m*, considering the corresponding likelihood:

4 $$y(t)={\beta }_{0}+{\beta }_{1}{\int }^{}\hat{h}(m)p(m|t)dm$$

where *y*(*t*) is the predicted contrast threshold at time *t*, and ${\beta }_{0}$ and ${\beta }_{1}$ are free parameters to scale the prediction of the model to the data.

**Figure 6:**
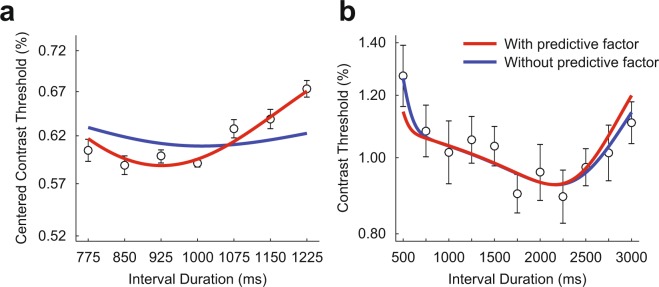
The lines of best fit for two generative models. The models are based on a subjective hazard function which involves probabilistic computations of the elapsed time and the probability of missing the target. The final model (red curves) assumes that attention level at a given moment reflects the probability that a target will appear at that instant or in the near future. Only the final model provides a good fit for the data of both experiments, with reasonable best-fitting parameter values (*w* = 0.11, *μ* = 106 ms).

We first fitted a model that does not consider the aforementioned probability of future event to the contrast thresholds in Experiment 1 and 3. A common Weber fraction parameter, *w*, was applied to the data for Experiment 1 and 3 assuming that the Weber fraction would be constant across different experimental conditions. The model resulted in a poor fit particularly for the data of Experiment 1, which was conducted with the narrower range of interval durations (blue curves in Fig. 6).

Thereafter, we fitted a model that assumes the level of attention at a given moment is modulated not only by the probability of the target appearing at that moment, but also by the probability of the target appearing near after that moment. An exponential weight function was used to quantify the degree by which the probability of a future event affects the current level of attention. The hazard function that implements the idea of predictive attention, ${h}_{{\rm{pred}}}(t)$, can be formulated as:

5 $${h}_{{\rm{pred}}}(t)=\frac{\frac{1}{\mu }{\int }_{t}^{\infty }f(\tau ){e}^{\frac{-(\tau -t)}{\mu }}d\tau }{1-kF(t)}$$

where $\mu$ is a free parameter characterizing the weights on future events, which is assumed to decrease exponentially. We substituted ${h}_{{\rm{pred}}}(t)$ for $h(t)$ in Eq. (3) and then fitted the model to the data. The model provides a better fit for the data of both Experiment 1 and 3 (red curves in Fig. 6) than the model without considering the predictive attention (*F*_1,12_ = 10.51, *p* = 0.007; chi-square difference test). The best-fitting value of Weber fraction parameter (*w* = 0.11) was in the range of the previously reported Weber fractions for time estimation^37–39^, and the best-fitting value of predictive term $\mu$ was 106 ms.

Apparently, the gradual increase of contrast threshold (i.e. decreasing performance) in a narrow range of ISIs predicted by the suggested model does not seem to be consistent with the results of existing studies that follow hazard function, in which the perceptual performance increases as ISI increases^23,24^. In fact, our model does not contradict the existing findings. According to the simulation results of our model, the pattern of temporal dynamics depends on the size of sensory noise, the probability of missing the target, and the range of ISIs, in addition to the hazard rate. The effect of predictive attention is minimal when the range of ISIs between the cue and target is relatively wide (e.g. 3000 ms). Consequently, when applied to a condition with wider range of ISIs and negligible missing probability, our model predicts monotonically decreasing thresholds (i.e. increasing performance) as a function of ISI mirroring the results of existing studies^23,24^.

## Discussion

In everyday life, we must often prepare ourselves to concentrate temporally on an upcoming event. Referees in a baseball game must focus their attention on the moment at which a runner is tagged by a baseman, and racecar drivers must focus their attention on a starter pistol at the beginning of a race. To effectively process relevant information in such situations, the perceptual system should allocate attention properly across the temporal domain. Maintaining a high level of attention for an extended duration would not be efficient. Paying attention only to a brief moment of an event is difficult, because it is implausible to estimate the time of a future event precisely and to control attention accordingly with infinite temporal resolutions. Therefore, the allocation of temporal attention across time should reflect the attention system’s strategy to deal with temporal uncertainty of an event and limited mental capacity^40^.

We measured the contrast sensitivity in orientation discrimination task to infer the temporal dynamics of visual attention. It was found that temporal attention is constant until the attention-induced moment of an event, and decreases afterward. A control experiment ably ruled out the possibility that perceptual bias in time estimation caused the observed pattern. In a further experiment with a wider range of cue-stimulus-intervals, we observed that attention level increased until the last 500 ms of the interval range, and started to decrease.

Results are not consistent with the known characteristics of the exogenous or the endogenous temporal attention. Studies of the temporal entrainment, a representative example of the exogenous attention, showed that the level of temporal attention peaked at a moment when an entrainer was to appear, and symmetrically decreased around the moment^10,41^. However, there are notable differences between those studies and the current study. Studies of visual temporal entrainment^10^ used significantly shorter interval durations between entrainers (≤400 ms) than our study (1000 ms). Although there are studies of auditory temporal entrainments^41^ that used relatively longer interval durations (~600 ms), those results are difficult to be directly compared to the current results due to differences in modality (visual vs. auditory) and experimental paradigm (simple detection task vs. delayed discrimination task). Our results suggest that the interval duration between entrainers used in our task was too long to effectively induce a stimulus-driven temporal entrainment for a visual task.

Our results seem to deviate from the characteristics of the endogenous temporal attention, which is modulated by conditional probability, as well. Contrary to the results of Experiment 1, it has been shown that the attention level roughly followed the hazard rate^16–24^. There were two differences between the existing studies manifesting the hazard rate and Experiment 1. First, the missing probability, which is the probability of missing a target when the target appeared, was negligible in existing studies, while it was around 0.5 in Experiment 1. Second, the range of ISIs were relatively longer in existing studies (longer than 2500 ms) compared to Experiment 1 (600 ms). Simulation results of our model suggest that both missing probability and the range of ISIs are important factors for shaping the pattern of temporal dynamics.

The hazard rate is not a realistic model of the conditional probability that subjects can actually calculate. First, the hazard rate assumes that subjects can access the current time precisely, however human time perception is unavoidably contaminated by sensory noise. Any realistic model of subjective hazard rate should consider the sensory noise, which will reshape the prediction of the model. Second, the hazard rate is calculated assuming that the probability of missing a presented target is zero. In most of the existing studies, the missing probability might have been negligible. However, it is critical to consider the missing probability to appropriately model the conditional probability when the missing probability is significantly above zero, as in the current experiments. Third, our results show that a model with a predictive attention can fit to the empirical data better, especially when the range of ISIs is relatively narrow. The model assumes that attention level will be modulated by the probability of target appearance not only at the current moment, but also in the near future. It is plausible that the predictive attention modulation is a strategy to compensate for the lag in temporal control of visual attention^42^.

By taking into account the aforementioned three factors, we developed a more realistic model of conditional probability that subjects calculated. The pattern of temporal attention predicted by the suggested model, a subjective hazard rate model, is qualitatively different from the prediction of the conventional hazard rate (see red curves in Fig. 6). The difference is especially apparent when the range of ISIs is narrow and the missing probability is high, as in Experiment 1.

To effectively deal with a large amount of visual input with limited mental capacity, the visual system must allocate attentional resources properly across temporal and spatial domains. In the current study, we directly examined temporal dynamics of visual attention allocation around an attention-induced moment with two different ranges of ISIs. We developed the subjective hazard rate model to quantitatively simulate the performances of the visual attention system observed in Experiments 1 and 3. Simulation results suggest that the temporal dynamics of visual attention is consistent with the performances of the subjective hazard rate model that takes into account the realistic conditional probability of an event in the current moment and in the near future.

## Methods

### Experiment 1

#### Subjects

Fifteen subjects (five females and ten males) participated in Experiment 1. Their mean age was 21.6 years, with a standard deviation of 1.76 years. All subjects were naive to the purpose of the study and had normal or corrected-to-normal vision. They signed an informed consent form approved by the Institutional Review Board (IRB) of Ulsan National Institute of Science and Technology (UNIST) and were compensated for their time. All methods were carried out in accordance with relevant guidelines and regulations.

#### Apparatus

Stimuli were created in MATLAB and the Psychophysics Toolbox^43,44^, and were shown on a DLP projector (PROPixx; 1920 × 1080; 120 Hz; linear gamma). Background luminance was 68.65 cd/m^2^. The viewing distance was 1.3 m.

#### Stimuli and design

Throughout the experiment, the cue and target stimulus appeared at the center of a uniform gray background. The target stimulus was a Gabor patch (a sinusoidal grating of one cycle per degree enveloped by a Gaussian window; 3σ; 10° × 10°). When the RGB number assigned to a certain pixel was not an integer, the number was rounded up or down probabilistically based on the number below the decimal point. This technique was used to obtain an integer for an RGB value while taking into account its fraction digits. The stimulus was tilted 45° either to the left or to the right, and its contrast range was set differently for each subject, based on their performance in a preliminary experiment. The presentation order of the orientation and contrast of the Gabor patch were randomized from trial to trial.

Before the main experiment, a preliminary experiment was conducted to determine a suitable contrast range for each subject. In the preliminary experiment, an empty circle (0.88° × 0.88°) appeared and shrank for 200 ms to become a dot that lingered for another 200 ms to cue the target appearance. Following an inter-stimulus interval of 300 ms, the Gabor stimulus appeared for 20 ms, and its contrast varied from a Michelson contrast of 0.1% to 2% in 25 log increments, regardless of subject.

In the main experiment, the stimulus was cued by a five-second countdown before it appeared. Arabic numerals (2.34° × 1.54°) appeared in descending order, from 5 to 1, for 50 ms each and with 1000 ms of stimulus-onset asynchrony (SOA), and then the Gabor stimulus appeared for 20 ms. At this point, SOA between the number “1” and the Gabor stimulus was randomly varied with equal probability from 700 ms to 1300 ms in increments of 75 ms (Fig. 2). This interval duration acted as an independent variable in the experiment. The stimulus contrast was varied from an estimated contrast of a 52.5% correction rate (*M* = 0.31%, *SD* = 0.08; in Michelson contrast) to that of a 97.5% correction rate (*M* = 0.95%, *SD* = 0.22) in 10 log increments, which were obtained by fitting a psychometric function to each subject’s results in the preliminary experiment.

#### Procedure

Subjects completed two blocks (500 trials each) for the preliminary experiment, which lasted approximately 1 h. For the main experiment, subjects completed four blocks (225 trials each) over two days, which lasted 2 h in total. Every block had ten practice trials in the beginning. In a trial, subjects sat in a comfortable position in a dark room, with their chin on a chin rest, looking at the center of a screen with binocular vision. They were asked to keep their eyes on the center of the screen. Instructions were provided in written form.

In a trial, subjects performed a two-alternative forced-choice (2AFC) orientation discrimination task. They reported the orientation of the Gabor patch stimulus by pressing either a left arrow key (leftward tilt) or a right arrow key (rightward tilt).

#### Data analysis

From each subject, contrast thresholds for nine interval durations were obtained by fitting psychometric functions to the 2AFC data and estimating contrast value at a 75% correction rate. There were nine parameters for the thresholds of nine interval durations, and one parameter for the slope of all intervals, assuming that the slope for a subject did not vary significantly across intervals. After acquiring nine contrast thresholds for each subject, we statistically assessed the existence of systematic short-term fluctuation of thresholds on individual levels, which can represent temporal oscillation of attention^45^. We used a permutation test that repeatedly computes standard deviation of differences in contrast thresholds between two successive interval durations 10,000 times, shuffling the data labels of interval duration on each iteration. The exchangeability requirement for a permutation test is met because under the null hypothesis of no systematic short-term fluctuation, the *SD* of threshold differences between neighboring interval durations is purely due to a random noise around long-term trends; thus, it would not be expected to differ after shuffling interval duration labels. Each individual subject showed no significant fluctuation, at least not short-term, to be shown in a 75 ms interval (*p* > 0.05 for all subjects; one-sided permutation test). Given that there is no systematic short-term fluctuation, a moving average across three adjacent thresholds was applied to smooth out short-term noises and emphasize overall trends. Finally, for each subject, mean threshold values were subtracted from thresholds to emphasize systematic changes in them across interval durations, and the overall mean of thresholds was added to the thresholds so that they would be centered at the mean. When performing statistical analysis, in addition to ANOVA, pairwise comparison using Fisher’s least square difference procedure was conducted to examine the differences in contrast thresholds between pairs of interval durations.

### Experiment 2

#### Subjects

The same group of 15 subjects who participated in Experiment 1 were recruited for Experiment 2A, and eight subjects (two females and six males), including two newly recruited subjects, participated in Experiment 2B. All subjects were naive to the purpose of the study and had normal or corrected-to-normal vision. They signed an informed consent approved by UNIST’s IRB and were compensated for their time. All methods were carried out in accordance with relevant guidelines and regulations.

#### Stimuli and design

Stimuli were identical to those of Experiment 1, except for the contrast of target stimulus. Since we were interested in subjects’ time judgment, contrasts were enhanced to minimize trials in which subjects would not notice the appearance of a target stimulus. By doing so, we could estimate the physical duration perceived as one second more efficiently. The contrast range was set differently for each subject, based on their performance in a preliminary experiment. In Experiment 2A, stimulus contrast was varied from an estimated contrast of an 85% correction rate (*M* = 0.78%, *SD* = 0.17) to that of a 97.5% correction rate in 10 log increments, which were obtained by fitting a psychometric function to each subject’s results in the preliminary experiment. In Experiment 2B, which was conducted to examine the effect of contrast on perceived duration, the stimulus contrast was set higher than in Experiment 2 A. The stimulus contrast was set to an estimated contrast of a near 100% correction rate (*M* = 2.19%, *SD* = 0.39). The interval durations used in Experiment 2A and 2B were identical to those of Experiment 1.

#### Procedure

Experiment 2A and 2B were identical in experimental procedure. Each subject ran two blocks of 180 trials, which lasted approximately one hour. Each block had ten practice trials in the beginning. The time course of a trial (Fig. 2) was identical to that of Experiment 1, except for responses. Subjects were asked to report whether the perceived interval duration between the last countdown and the target was longer than one second or not, after reporting the orientation of a target grating. Subjects pressed the up arrow (longer), down arrow (shorter), or “0” (fail to detect) keys to respond. Trials with “fail to detect” responses were removed prior to further analysis.

#### Data analysis

Subjects’ biases in the duration judgment task were estimated by fitting psychometric functions to their data. We applied a hierarchical Bayesian model to estimate the thresholds and slopes of the psychometric functions. Specifically, we assumed that the thresholds and slopes for each subject were drawn from independent Gaussian distributions representing population distributions of parameters. Priors on the means and *SD*s of the population distributions were set to uniform distributions with ranges large enough to cover all practically possible values. We used a Metropolis-Hastings algorithm to directly sample from posterior distributions. The reason for using a hierarchical Bayesian model was to improve the reliability of parameter estimation. Several subjects in Experiment 2 showed strong biases in perceived durations that were beyond the range of presented interval durations. In such cases, the estimated parameters were unstable. A benefit of using the hierarchical Bayesian model is that we could directly estimate the distributions of population parameters, which are reported above.

### Experiment 3

#### Subjects

A new group of 15 subjects (four females and eleven males) participated in Experiment 3. Their mean age was 22.1 years, with a standard deviation of 2.57 years. All subjects were naive to the purpose of the study and had normal or corrected-to-normal vision. They signed an informed consent form approved by UNIST’s IRB and were compensated for their time. All methods were carried out in accordance with relevant guidelines and regulations.

#### Stimuli and design

Stimuli and design were identical to those of Experiment 1, except for the following changes. The key difference was that in Experiment 3 we varied the SOA between the cue and stimulus with a wider range from 500 ms to 3000 ms.

As we recruited a new group of subjects and used a different range for interval duration, a preliminary experiment was conducted again to determine a suitable contrast range for each subject. The cue in the preliminary experiment was same with the one in the preliminary experiment of Experiment 1, but the SOA between the cue and the Gabor stimulus was randomly varied with equal probability from 500 ms to 3000 ms in increments of 250 ms. Regardless of the SOA between the cue and target, a black central dot (0.12° × 0.12°) appeared 3200 ms after the cue to instruct subjects to make a response. The contrast of the Gabor was varied from a Michelson contrast of 0.3% to 3% in 25 log increments regardless of subject.

In the main experiment, Arabic numerals appeared in descending order from 2 to 1 before the Gabor appeared. The SOA between the number “1” and the Gabor was again randomly varied with equal probability from 500 ms to 3000 ms, in increments of 250 ms, and the black dot appeared again 3200 ms after the number “1” appeared. The stimulus contrast varied from an estimated contrast of a 52.5% correction rate (*M* = 0.99%, *SD* = 0.86) to that of a 97.5% correction rate (*M* = 1.77%, *SD* = 0.80) in 10 log increments, which were obtained by fitting a psychometric function to each subject’s results in the preliminary experiment.

#### Procedure

Subjects completed five blocks (110 trials each) for the preliminary experiment, which lasted approximately 1 h. For the main experiment, subjects completed ten blocks over two days, which lasted 2 h in total. Every session had ten practice trials in the beginning. In each trial, subjects performed the same 2AFC task as in Experiment 1, except they were instructed to respond only after the presentation of the black central dot, which appeared 3200 ms after the cue appearance.

#### Data analysis

Since appropriate psychometric function fit to the individual subject’s data could not be obtained in some cases, we instead examined correction rates on individual level and contrast thresholds on group level. Both were qualitatively similar to each other, except that they were flipped upside down (i.e. higher level of attention results in higher correction rates and lower contrast thresholds). For the statistical analysis, we used correction rates as they allow analysis on individual levels (Fig. S1). For plotting, we used contrast thresholds to provide consistency with the results of Experiment 1. As done for the contrast thresholds in Experiment 1, for each subject, the mean correction rates were subtracted from correction rates to emphasize systematic changes in them across interval durations, and the overall mean of correction rates was added to the correction rates so that they would be centered at the mean. When performing statistical analysis, in addition to ANOVA, pairwise comparison using Fisher’s least square difference procedure was conducted to examine the differences in correction rates between pairs of interval durations. Contrast thresholds for eleven interval durations were obtained by fitting psychometric functions to the group 2AFC data and estimating contrast value at a 75% correction rate. Error bars were computed by bootstrapping the psychometric function fit 10,000 times, sampling from the subjects’ data with replacement on each iteration, and taking the *SD* of the contrast thresholds from the resulting bootstrapped distribution.

## Supplementary information

Supplementary information

## Data Availability

The datasets generated and analyzed during the current study are available from the corresponding author on reasonable request.
