# Supplementary material for: Temporal Dynamics of Visual Attention Allocation
Source: Sci Rep. 2019 Mar 6;9:3664. doi: 10.1038/s41598-019-40281-7 (PMC6403260; doi:10.1038/s41598-019-40281-7)
Supplement: Supplementary file 1 — Supplementary information [file 41598_2019_40281_MOESM1_ESM.pdf]

Supplementary Information

Temporal Dynamics of Visual Attention Allocation

Jongmin Moon[1a], Seonggyu Choe[1a], Seul Lee[1], and Oh-Sang Kwon[1*]

[1]Department of Human Factors Engineering, Ulsan National Institute of Science and Technology, Ulsan 44919, Republic of Korea

[a]J.M. and S.C. contributed equally to this work.

*Corresponding author: O.-S.K. (oskwon@unist.ac.kr)

Modeling

We can derive the hazard rate by considering the probability of the target not being detected even though it has already appeared:

$$h(t) = \frac{f(t)\big(1 - P(A|ND)\big)}{1 - F(t)} \tag{6}$$

where $P(A|ND)$ is the probability that the target has been missed (i.e., target has appeared but was not detected). $P(A|ND)$ was obtained by

$$P(A|ND) = \frac{P(ND|A)P(A)}{P(ND|NA)P(NA) + P(ND|A)P(A)} = \frac{0.5 \cdot F(t)}{1 \cdot \big(1 - F(t)\big) + 0.5 \cdot F(t)} \tag{7}$$

where $P(A)$ and $P(NA)$ are the probability that the target has appeared and has not appeared, respectively, and $P(ND|A)$ and $P(ND|NA)$ are the probability that the target is not detected given that it appeared and did not appeared, respectively. With equation (7), we can re-write the equation (6) to get equation (1).

Our model infers the expected value of the hazard rate at time *t*, given a sensory measurement *m*, and the target not being detected yet, which requires computations of $p(t|m, ND)$. By assuming the independence between the measurement of the elapsed time and failure to detect the target, the probability density can be computed as follows:

$$p(t|m, ND) = \frac{p(m, ND|t)p(t)}{\int p(m, ND|t)p(t)dt} = \frac{p(m|t)p(ND|t)}{\int p(m|t)p(ND|t)dt} \tag{8}$$

where $p(t)$ can be dropped off since we assumed no prior preference on time t, and $p(ND|t) = 1 - kF(t)$, just as the denominator in equation (1), which allowed us to simplify the notation in equation (3).

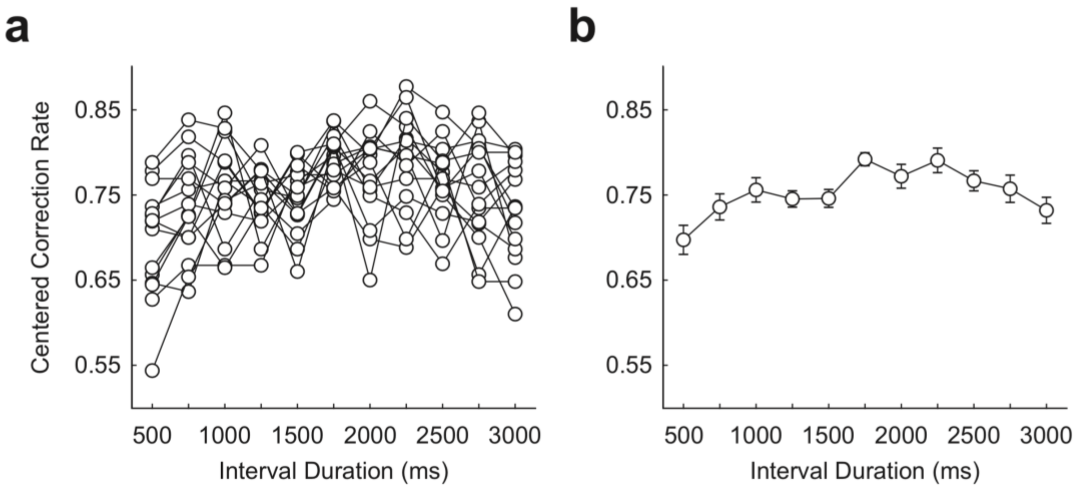

Figure S1 (Related to Fig. 5). Results of Experiment 3. We plot correction rates of (a) 15 subjects and (b) their means as a function of interval durations. They are qualitatively similar to the contrast threshold function (Fig. 5), except that they are flipped upside down. The error bars represent standard errors of the means (n = 15).

Interval duration (ms)		Mean diff. (95% CI)	*p*-value
775	850	.03 (–.02, .07)	.260
	925	.01 (–.04, .06)	.642
	1000	.02 (–.02, .07)	.343
	1075	–.04 (–.08, .01)	.104
	1150	–.05 (–.10, –.01)	.019
	1225	–.11 (–.15, –.06)	.000
850	925	–.02 (–.06, .03)	.507
	1000	.00 (–.05, .04)	.858
	1075	–.06 (–.11, –.02)	.007
	1150	–.08 (–.13, –.04)	.001
	1225	–.13 (–.18, –.09)	.000
925	1000	.01 (–.03, .06)	.627
	1075	–.05 (–.09, .00)	.038
	1150	–.07 (–.11, –.02)	.005
	1225	–.12 (–.16, –.07)	.000
1000	1075	–.06 (–.11, –.01)	.011
	1150	–.08 (–.12, –.03)	.001
	1225	–.13 (–.18, –.08)	.000
1075	1150	–.02 (–.06, .03)	.461
	1225	–.07 (–.12, –.02)	.003
1150	1225	–.05 (–.10, –.01)	.024

Table S1 (Related to Fig. 3). Results of pairwise comparison in Experiment 1.

	500	750	1000	1250	1500	1750	2000	2250	2500	2750	3000
500		.057	.004	.018	.017	.000	.000	.000	.001	.003	.087
750	–.04<br>(–.08, .00)		.322	.644	.620	.006	.076	.007	.130	.291	.843
1000	–.06<br>(–.10, –.02)	–.02<br>(–.06, .02)		.597	.620	.076	.428	.087	.597	.947	.235
1250	–.05<br>(–.09, –.01)	–.01<br>(–.05, .03)	.01<br>(–.03, .05)		.974	.022	.187	.026	.291	.552	.509
1500	–.05<br>(–.09, –.01)	–.01<br>(–.05, .03)	.01<br>(–.03, .05)	.00<br>(–.04, .04)		.024	.199	.028	.306	.574	.488
1750	–.09<br>(–.13, –.05)	–.06<br>(–.10, –.02)	–.04<br>(–.08, .00)	–.05<br>(–.09, –.01)	–.05<br>(–.09, –.01)		.322	.947	.210	.087	.003
2000	–.07<br>(–.11, –.03)	–.04<br>(–.08, .00)	–.02<br>(–.06, .02)	–.03<br>(–.07, .01)	–.03<br>(–.07, .01)	.02<br>(–.02, .06)		.355	.791	.467	.049
2250	–.09<br>(–.13, –.05)	–.05<br>(–.09, –.01)	–.03<br>(–.07, .01)	–.05<br>(–.09, –.01)	–.04<br>(–.08, .00)	.00<br>(–.04, .04)	–.02<br>(–.06, .02)		.235	.100	.004
2500	–.07<br>(–.11, –.03)	–.03<br>(–.07, .01)	–.01<br>(–.05, .03)	–.02<br>(–.06, .02)	–.02<br>(–.06, .02)	.03<br>(–.01, .07)	.01<br>(–.03, .05)	.02<br>(–.02, .06)		.644	.087
2750	–.06<br>(–.10, –.02)	–.02<br>(–.06, .02)	.00<br>(–.04, .04)	–.01<br>(–.05, .03)	–.01<br>(–.05, .03)	.03<br>(–.01, .07)	.01<br>(–.03, .05)	.03<br>(–.01, .07)	.01<br>(–.03, .05		.210
3000	–.03<br>(–.07, .01)	.00<br>(–.04, .04)	.02<br>(–.02, .06)	.01<br>(–.03, .05)	.01<br>(–.03, .05)	.06<br>(.02, .10)	.04<br>(.00, .08)	.06<br>(.02, .10)	.03<br>(–.01, .07)	.03<br>(–.01, .07)	

Table S2 (Related to Fig. 5 and Fig. S1). Results of pairwise comparison in Experiment 3. Headers represent interval durations (in ms), numbers in the lower triangular matrix represent mean differences in correction rates between pairs of interval durations and their 95% confidence intervals, and numbers in the upper triangular matrix represent the corresponding *p*-values.
